# Post Hoc Subgroup Analysis of the BCause Study Assessing the Effect of AbobotulinumtoxinA on Post-Stroke Shoulder Pain in Adults

**DOI:** 10.3390/toxins14110809

**Published:** 2022-11-20

**Authors:** Marcelo Riberto, João Amaury Frances, Regina Chueire, Ana Cristina Ferreira Garcia Amorim, Denise Xerez, Tae Mo Chung, Lucia Helena Costa Mercuri, Sérgio Lianza, Eduardo Carvalho de Melo Rocha, Pascal Maisonobe, Thais Cuperman-Pohl, Patricia Khan

**Affiliations:** 1 Faculdade de Medicina de Ribeirão Preto, Universidade de São Paulo, São Paulo 14049-900, Brazil; 2 Hospital Bettina Ferro de Souza, Campus IV da Universidade Federal do Pará, Belém 66075-110, Brazil; 3 Faculdade de Medicina de São José do Rio Preto, Autarquia Estadual 15090-000, Brazil; 4 Centro de Reabilitação e Readaptação Dr. Henrique Santillo (CRER), Goiânia 74653-230, Brazil; 5 Serviço de Medicina Física e Reabilitação, Hospital Universitário Clementino Fraga Filho, Universidade Federal do Rio de Janeiro, Rio de Janeiro 21941-590, Brazil; 6 Complexo Hospital das Clinicas, Instituto de Medicina Fisica e Reabilitação, São Paulo 04116-030, Brazil; 7 Hospital São Paulo—UNIFESP, São Paulo 04024-002, Brazil; 8 Hospital Alemão Oswaldo Cruz, São Paulo 01323-020, Brazil; 9 Serviço de Reabilitação da Irmandade da Santa Casa de Misericórdia de São Paulo, São Paulo 01221-010, Brazil; 10 Ipsen, 92100 Boulogne Billancourt, France; 11 Ipsen, São Paulo 04571-010, Brazil; 12 Centro Catarinense de Reabilitação, Florianópolis, Santa Catarina 88025-301, Brazil

**Keywords:** abobotulinumtoxinA, patients, post-stroke, shoulder pain, spasticity, therapeutic goal

## Abstract

**Abstract:**

Botulinum toxin type A is approved for the focal treatment of spasticity; however, the effectiveness of abobotulinumtoxinA (aboBoNT-A) in patients with shoulder pain who have set reduced pain as a treatment goal is understudied. In addition, some patients encounter delays in accessing treatment programs; therefore, the suitability of aboBoNT-A for pain reduction in this population requires investigation. These factors were assessed in aboBoNT-A-naive Brazilian patients in a post hoc analysis of data from BCause, an observational, multicenter, prospective study (NCT02390206). Patients (N = 49, *n* = 25 female; mean (standard deviation) age of 60.3 (9.1) years; median (range) time since onset of spasticity of 16.1 (0–193) months) received aboBoNT-A injections to shoulder muscles in one or two treatment cycles (*n* = 47). Using goal attainment scaling (GAS), most patients achieved their goal of shoulder pain reduction after one treatment cycle (72.1%; 95% confidence interval: 57.2–83.4%). Improvements in GAS T-score from baseline, clinically meaningful reductions in pain score at movement, and clinically meaningful increases in passive shoulder abduction angle further improved with repeated treatment more than 4 months later, despite treatment starting at a median of 16.1 months after the onset of spasticity. These findings support the further investigation of aboBoNT-A injections in chronic post-stroke shoulder pain.

**Plain Language Summary:**

After a stroke, patients often experience shoulder pain, which can lead to difficulty with arm movement and interfere with their rehabilitation. Some patients also experience difficulty or delays in receiving treatment. Botulinum toxin injections can be used to improve muscle pain and function. The BCause trial looked at how one or two doses of a specific type of botulinum toxin injection, called abobotulinumtoxinA (aboBoNT-A), into shoulder muscles could help patients with shoulder pain following a stroke. This analysis of the BCause trial included 49 Brazilian patients aged 18–80 years who had suffered a stroke in the previous year and who set reduced shoulder pain as a goal of their treatment. They had not previously received any botulinum toxin injections. The researchers looked at how helpful the injections were by using a scale that measured how well each patient achieved their treatment goal. Additionally, researchers measured pain levels, muscle tone, range of shoulder movement, and quality of life before and after treatment. The results showed that aboBoNT-A injections helped most patients to reach their goal of reduced shoulder pain. Patients receiving repeated aboBoNT-A injections experienced further improvements in how much they could move their shoulder after more than 4 months compared with at the start of the study. Patients’ and caregivers’ quality of life was also improved after treatment compared with before. The researchers considered these results to be clinically meaningful—that is, the improvements with aboBoNT-A were likely to provide a real benefit for patients, caregivers, and clinicians.

## 1. Introduction

Shoulder pain is one of the most common post-stroke complications, with the reported incidence ranging from 5% to 84% [1,2,3,4,5,6]. Shoulder pain leads to a reduction in the shoulder joint range of motion (ROM), a decrease in the functional use of the arm, and restriction of patient performance and participation in rehabilitation [5,6,7,8].

Botulinum toxin type A (BoNT-A) injections are approved for the focal treatment of spasticity in adults and children (≥2 years of age) to improve muscle tone and function [9,10,11,12]. BoNT-As have also demonstrated analgesic properties in numerous therapy areas [13,14,15]. In a review of 19 clinical studies, intramuscular BoNT-A injections were reported to be helpful in patients with stroke who had hemiplegic shoulder pain [13]. In a prospective multicenter study in 60 patients with spasticity and pain, intramuscular BoNT-A injections were reported to reduce spasticity-related local pain [14]. Prospective, placebo-controlled, double-blind studies provide evidence for the efficacy of BoNT-A treatment in therapy areas including cervical dystonia, pelvic pain, low back pain, plantar fasciitis, postsurgical painful spasms, myofascial pain syndromes, migraine, and chronic daily headaches [15]. The underlying mechanisms behind the antinociceptive effects of BoNT-A are due to the inhibition of peripheral neurotransmitters, such as acetylcholine and inflammatory mediators released from motor neurons and autonomic synapses [16,17]. BoNT-A may also act at the spinal cord level to induce central antinociceptive activity through plastic rearrangement in the brain subsequent to denervation or alterations in sensory output [18,19]. Treatment with BoNT-A in patients with shoulder pain has been shown to provide clinically meaningful improvements in pain and mobility, with well-tolerated safety profiles across studies [20,21,22]. A randomized, double-blind, placebo-controlled study found that BoNT-A injected into the subscapularis muscle was beneficial in the management of shoulder pain in patients with post-stroke shoulder pain. Lateral rotation was also improved [20]. A systematic review and meta-analysis of randomized controlled trials comparing the clinical efficacy (pain intensity and shoulder range of motion) of BoNT-A injection with conventional therapy found that patients treated with BoNT-A had improved pain scores and shoulder abduction ROM versus those in the control group [21]. In a follow-up study of patients with refractory post-stroke shoulder pain, patients receiving BoNT-A injections plus rehabilitation had reduced pain during shoulder motion, mainly during the movements of extension and rotation versus baseline [22]. Treatment with abobotulinumtoxinA (aboBoNT-A, Dysport^®^, Ipsen, Paris, France) injections to shoulder muscles in adults with upper limb spasticity improved pain (−0.7; Disability Assessment Scale) and active function (+0.60; Modified Frenchay Scale score) in a phase 3 open-label study (NCT01313299) [23].

However, there are limited data regarding the effectiveness of aboBoNT-A for the treatment of shoulder pain in patient populations who have set reduced pain as their primary treatment goal. Goal achievement in patients with hemiplegic shoulder pain has been reported with BoNT-A treatment; however, the proportion of patients who set pain as the primary treatment goal was low (25.6%) [23]. Additionally, there is a need to investigate the attainment of pain reduction goals with BoNT-A in patients who encounter barriers and delays to treatment, for example, those in low- to middle-income countries, such as Brazil [24].

BCause was an observational, multicenter, prospective study (NCT02390206) designed to assess the effectiveness of BoNT-A treatment in Brazilian patients with chronic post-stroke (hemorrhagic or ischemic) spasticity affecting upper and lower limbs [25]. Of patients who received BoNT-A injections to the upper limb (*n* = 200), 57.8% were responders (defined as the achievement of the primary goal from the goal attainment scaling (GAS) at visit 2; primary endpoint). The upper limb mean GAS T-score (which indicates overall improvement (higher score) or worsening (lower score) of outcomes) increased by 14.13 from baseline during cycle 1 [25]. BoNT-A injections improved mobility (shoulder abduction angle; ROM), pain (pain score; visual numeric scale (VNS)), muscle tone (modified Ashworth scale (MAS) score), and independence (Barthel Index score) in these patients.

Here, we present a post hoc analysis of data from the BCause Study to assess the effect of aboBoNT-A in the management of chronic post-stroke shoulder pain in patients who set pain as a therapeutic goal.

## 2. Results

### 2.1. Participants

Overall, 239 patients were enrolled in the BCause Study [25]. Of these, 49 set shoulder pain as a primary or secondary treatment goal at their first and/or second injection and received aboBoNT-A injections to the shoulder; these patients were included in this post hoc analysis.

Baseline demographics are presented in Table 1. The mean (standard deviation (SD)) age of the patients was 60.3 (9.1) years, 51.0% were female, and 89.8% were right-handed. The median (range) time since the last cerebrovascular accident (CVA), onset of CVA, and onset of spasticity was 20.6 (12–240), 21.2 (12–240), and 16.1 (0–193) months, respectively. All the patients had spasticity that affected the upper limbs, and most (65.3%) had no or mild impairment of communication. The remainder had significant or total impairment (18.4%), or data were not available (16.3%). Overall, 12% of the patients were using oral antispasmodics, with baclofen, tizanidine, and diazepam use being recorded. All patients received injections in upper limbs at the first visit, with 47 of these receiving injections during cycle 2. One patient only received injections to lower limbs during cycle 2. Table 2 describes the muscles injected and doses used during cycles 1 and 2 of the study. The mean (SD) time between the first and second treatment was 4.63 (0.74) months. The most frequently injected muscle in cycle 1 was pectoralis major (89.8%), followed by latissimus dorsi (55.1%) and subscapularis (26.5%). Injection was most often guided by palpation/anatomic landmarks, a practice previously described among Brazilian healthcare providers [26].

### 2.2. Effectiveness

The majority of the patients achieved or overachieved their goal of shoulder pain reduction (responders, GAS of 0, +1, or +2). Of the patients with available data, 72.1% (95% confidence interval (CI): 57.2, 83.4; 31/43 patients) were responders after the first injection cycle and 60.9% (95% CI: 40.7, 77.9; 14/23 patients) were responders after the second injection cycle.

**Figure 1:**
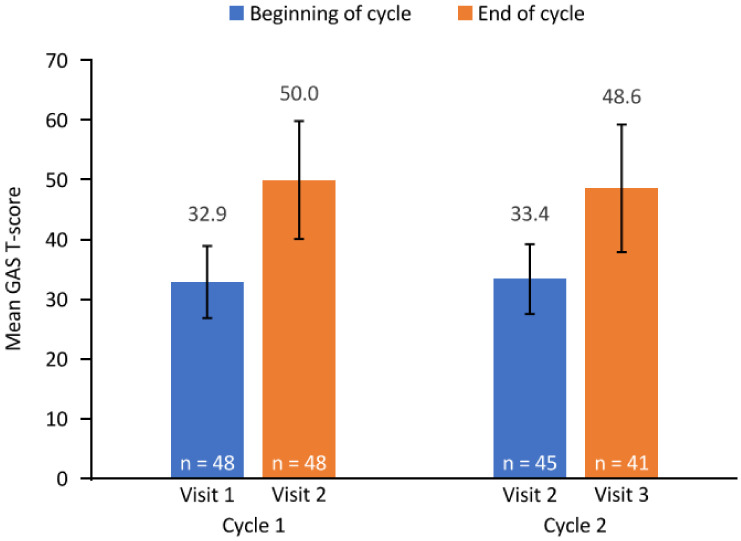
GAS T-score. Error bars depict standard deviation. The end of cycle 1 and beginning of cycle 2 assessments were both conducted at visit 2. Three patients completing cycle 1 did not receive a second cycle of injections. GAS, goal attainment scaling; n, number of patients with available data.

GAS T-scores improved by the end of each treatment cycle (Figure 1). The mean (95% CI) change from baseline in GAS T-score during cycle 1 (*n* = 48) was 17.1 (13.9, 20.4) and 14.9 (11.4, 18.4) during cycle 2 (*n* = 41). The mean (SD) overall cumulated GAS T-score (defined as the mean GAS T-score across both cycles or equal to the achievement score if only one cycle was completed; N = 49 (both cycles, *n* = 17; only cycle 1, *n* = 26; only cycle 2, *n* = 6)) was 49.6 (8.0).

**Figure 2:**
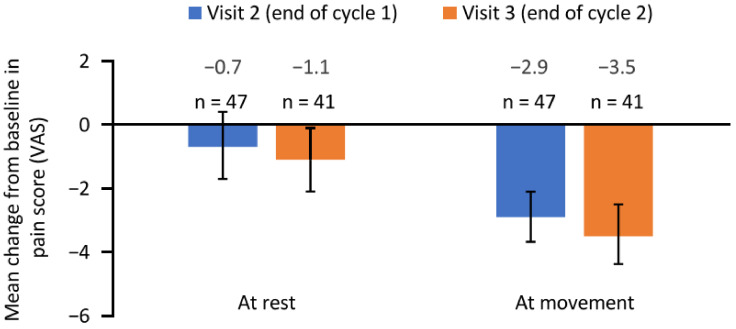
Change in pain score from baseline to the end of cycle 1 (visit 2) and the end of cycle 2 (visit 3). Error bars depict 95% confidence intervals. n, number of patients with available data; VAS, visual analogue scale.

Patients reported a reduction in pain following aboBoNT-A injections as measured using a visual analogue scale (VAS) (Figure 2). The mean (95% CI) change in pain score at movement was −2.9 (−3.7, −2.1) from baseline to the end of cycle 1 (visit 2) and −3.5 (−4.4, −2.5) from baseline to the end of cycle 2 (visit 3). The mean (95% CI) change in pain score at rest was −0.7 (−1.7, 0.3) from baseline to visit 2 and −1.1 (−2.1, −0.1) from baseline to visit 3.

**Figure 3:**
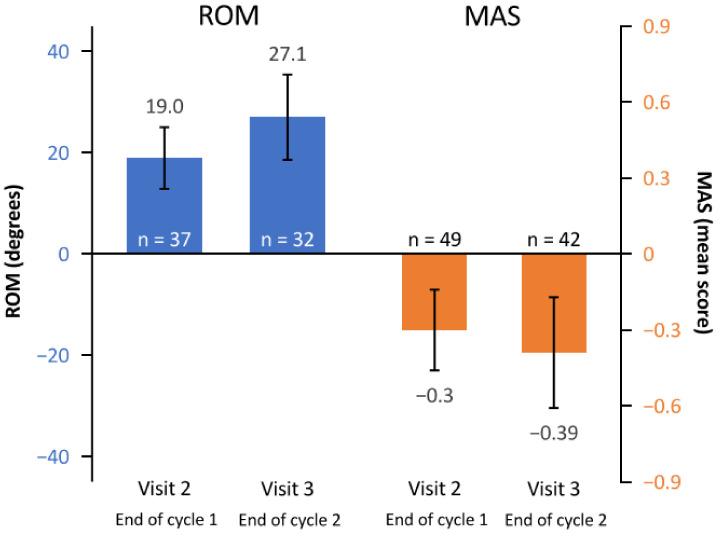
Mean change from baseline to the end of cycle 1 (visit 2) and the end of cycle 2 (visit 3) in ROM (shoulder abduction angle) and MAS (muscle tone). Error bars depict 95% confidence intervals. MAS, modified Ashworth scale; n, number of patients assessed; ROM, range of motion.

ROM, muscle tone, and passive function improved after each treatment cycle (Figure 3). In terms of ROM, the mean (95% CI) change in shoulder abduction angle from baseline was 19.0 (12.9, 25.1) degrees and 27.1 (18.7, 35.5) degrees by visits 2 and 3, respectively. The mean (95% CI) change in MAS score from baseline was −0.30 (−0.46, −0.14) and −0.39 (−0.61, −0.17) for visits 2 and 3, respectively. 

Quality of life (QoL) was improved following treatment with aboBoNT-A injections (Table 3). Patients became less dependent throughout the study, as measured by the Barthel Index score, and of those with data available from the five-dimension five-level EuroQol questionnaire (EQ-5D-5L), 44.0% (11/25) and 65.2% (15/23) of the patients reported at least one level of improvement from baseline in pain/discomfort at visits 2 and 3, respectively. At visit 2, a weak correlation (Spearman coefficient (95% CI)) between change from baseline in pain score at movement and change from baseline in shoulder MAS score was observed; this correlation was not statistically significant (*n* = 47; 0.23 (−0.06, 0.48)). At visit 3, no correlation between pain score and shoulder MAS score was observed (*n* = 41; 0.08 (−0.24, 0.37)).

At visits 2 and 3, very weak and nonsignificant correlations (Spearman coefficient (95% CI)) were observed between mean change from baseline in pain score at movement and change in ROM (*n* = 35; 0.04 (−0.30, 0.36) and *n* = 31; −0.03 (−0.38, 0.33), respectively) and EuroQol (EQ) VAS score (*n* = 24; −0.19 (−0.55, 0.23) and *n* = 23; −0.12 (−0.51, 0.30), respectively).

At the end of cycles 1 and 2, 77.6% (38/49) and 85.7% (36/42) of the patients, respectively, reported some or great benefit from the treatment. Corresponding values for physician-reported treatment benefits were 98.0% (48/49) and 92.9% (39/42), respectively.

Overall, 85.7% of the patients (42/49) required caregivers. In most cases (39/42; 92.9%), this was (or included) a family member, and in 51.3% of the cases (20/39), caregiving affected his/her professional activity. Caregivers’ physical and psychological burden of disease decreased from baseline in 76.2% (32/42) and 57.1% (24/42) of the cases, respectively, after cycle 1, and in 71.4% (25/35) and 65.7% (23/35) of the cases, respectively, after cycle 2.

## 3. Discussion

This post hoc analysis of data from the BCause Study demonstrated the effectiveness of aboBoNT-A injections in Brazilian patients with chronic post-stroke shoulder pain who set pain as a therapeutic goal. Improvements in GAS T-score from baseline, clinically meaningful reductions in pain score at movement, and clinically meaningful increases in passive shoulder abduction angle improved further with repeated treatment after more than 4 months. Improvements in patients’ QoL and reductions in caregiver burden were also observed. The magnitude of change from baseline in pain score was not significantly associated with levels of improvement in muscle tone, mobility, or QoL in this post hoc analysis. The improvements seen with aboBoNT-A are in line with a trend of successful treatment noted with the use of a recently developed diagnostic algorithm [27]. In the algorithm, diagnostic nerve block was used to assess early-phase hemiplegic shoulder pain and to guide clinicians into selecting the most appropriate diagnosis of the etiology of pain, with BoNT-A being effective in the case studies when the algorithm was applied.

The etiology of post-stroke shoulder pain is multifactorial and includes soft tissue disorders (rotator cuff lesions, glenohumeral dislocation, hand–shoulder syndrome, myofascial pain syndrome, spasticity and contractures, adhesive capsulitis) that cause peripheral and central pain sensitization due to localized ischemia and release of algogenic substances [2,6,28,29]. As well as reducing muscle tone, aboBoNT-A may inhibit the release of local nociceptive neuropeptides to reduce pain sensitization [16]. In the previously published analysis of BCause, aboBoNT-A was shown to reduce pain in the overall population [25], and this treatment benefit is confirmed in the ad hoc analysis reported here in patients with a goal of pain reduction. Pain reduction, together with improved mobility, ROM, and motor function, is a frequent goal of therapy and was identified as a primary or secondary goal by 21% of the BCause population. It was beyond the scope of this analysis to compare outcomes in patients who had and had not identified pain as a key goal, but this could be a focus of future analysis. Understanding more about the underlying etiology of shoulder pain in patients post-stroke would also be valuable. Shoulder pain is a frequent concern in patients in the over-60-years age group irrespective of stroke. As an observational study, a history of shoulder pain prior to the stroke was not recorded, and it is possible that some patients were experiencing impingement syndrome or other pathologies unrelated to stroke. Future analyses should consider collecting additional information on patient history prior to stroke to allow a more detailed picture to emerge.

In the BCause Study, the patients received injections into the adductor muscles of the shoulder (pectoralis major, subscapularis, teres major, latissimus dorsi), and the results, therefore, support the muscular etiology of the pain, originating from the joint movement disorganization mediated by adductor–abductor imbalance [19]. Indeed, in a previous study of post-stroke survivors from Brazil, increased adductor tone was a frequent finding [30], also noted in patients with spastic hemiplegia [21,22]. Consistent with clinical practice in Brazil [26], anatomic palpation was used to guide injection in most patients; however, it is important to acknowledge that this may not be routine practice in other countries where electrical stimulation or ultrasound may be more widely available and, therefore, more commonly used to guide injections [31,32].

Results presented here reflect those of other real-world studies of BoNT-A treatment for upper limb spasticity [33,34]. In the Upper Limb International Study (ULIS)-II and ULIS-III, respectively, 32% and 40% of the patients received injections to shoulder muscles, and pain reduction was a goal for treatment in 31% (*n* = 145) and 37% (*n* = 349) of the patients [33,34]. After one treatment cycle, 84% and 66% of these patients, respectively, were responders, which is comparable to the 72% response rate in the present study [33,34]. The mean GAS T-score was 52.0 (weighted (SD 10.1)) in ULIS-II and 49.8 (overall (95% CI: 49.2, 50.3)) in ULIS-III, with a mean (SD) change from baseline of 17.6 (11.0; *p* < 0.001) and 13.1 (not reported), respectively [33,34]. Similarly, in our post hoc analysis, the mean (SD) overall cumulated GAS T-score was 49.6 (8.0), with a mean (SD) change from baseline of 17.1 (11.3) during cycle 1 and 14.9 (11.0) during cycle 2.

Here, the patients reported clinically relevant lower pain scores (median −1.1) at visit 3 (end of cycle 2) than at baseline. This is consistent with results from previous trials. For example, in a study of patients with post-stroke upper limb spasticity, a trend for reduced pain over the first injection cycle was observed; however, this result was not statistically significant [35]. In another study, in which patients with upper limb spasticity received repeated cycles of aboBoNT-A, there was an improvement in passive and active ROM in the shoulder extensors at week 4 of each cycle, and passive motion improvements increased between cycles [36]. Furthermore, passive and active function were improved with aboBoNT-A 1500 U compared with 1000 U, because 1500 U included shoulder injections, whereas 1000 U did not. This may indicate the importance of shoulder muscle injections, possibly as a result of improved active shoulder flexion, which is a joint movement required in most daily activities [36].

In terms of passive ROM, the findings for ULIS-II and the present analysis highlight the potential clinical benefit of treatment with BoNT-A in patients with chronic post-stroke upper limb spasticity [33]. The increase in passive ROM and decrease in pain in the upper limb at active movement shown here may be due to the fact that adductor muscles of the shoulder were injected with aboBoNT-A in this study. These findings are consistent with several studies that have investigated the use of BoNT-A in the reduction of pain and improvement of ROM in the shoulder [20,22,37]. Interestingly, greater pain reduction was reported at active movement (i.e., during daily activities) than at rest in the current study. However, the correlation between change from baseline in pain score at movement and ROM was very weak and not statistically significant. This result does not align with the findings of a large meta-analysis that reported the effectiveness of BoNT-A for the treatment of shoulder pain [21]; the lack of statistical significance observed in the current analysis could be driven by the small sample size.

Furthermore, in ULIS-II and ULIS-III, respectively, the mean MAS total score at follow-up (3–4 months after one treatment cycle) was 8.4 (SD: 3.4) and 9.4 (95% CI: 9.1, 9.6), with a mean change from baseline of −2.6 (95% CI: −2.9, −2.4; *p* < 0.0001) and −0.5 (95% CI: −0.6, −0.4; *p* < 0.0001). Both the findings in the ULIS studies and the present analysis highlight the potential clinical benefit of treatment with aboBoNT-A injections to improve muscle tone in this patient population [33,34].

The reported independence of the patients increased throughout the study, demonstrated by an increased Barthel Index score and reduced physical burden on caregivers. This is consistent with literature highlighting the potential of treatment with aboBoNT-A injections to improve the independence of adults with upper limb spasticity [33,34]. In the study previously described of patients with upper limb spasticity who received repeated cycles of aboBoNT-A, scores for dressing, hygiene, and pain improved progressively across cycles, paralleled by small but positive changes in QoL [36]. Following aboBoNT-A injections, QoL was also improved in the current study and in previous trials of post-stroke upper limb spasticity [35,38].

A sustained treatment effect was observed throughout the study. Patients and caregivers reported increased independence and reduced disease burden, respectively, at the end of both cycles. This may potentially reduce the amount of care that is required to be provided to patients by their respective caregivers. It may also reduce distress caregivers may experience when they are helping with activities, such as dressing and cleaning, that cause the patient pain.

These findings could potentially address an unmet clinical need in this patient population. Patients often experience delays in admission to a rehabilitation program in Brazil following a stroke [39]. Here, pain relief was reported following aboBoNT-A treatment despite patients being treated at a median of 16 months after the onset of stroke or spasticity. It is feasible that a greater magnitude of pain relief and improvement in ROM may have been reported if treatment had been initiated earlier (within 6 months of a stroke) for patients in the present study [39]. However, the patients did not return to baseline levels before retreatment at visit 2, and an enhanced effect was observed upon repeat treatment; therefore, repeat injections may overcome any treatment lag effect. The results from this analysis demonstrate the potential for BoNT-A treatment in the management of post-stroke pain; however, head-to-head comparisons with other therapies are currently lacking, and these would be valuable in this area to establish how best to optimize pain management in this patient population.

The current analysis was limited by both being a post hoc analysis and using a small sample size; however, because this analysis examined a specific patient group within a study, this was expected. Other limitations reflect the observational nature of the BCause Study. Information on the exact injection points of the muscles was not collected, and clinicians were free to choose the injection muscles in accordance with the local summary of product characteristics and locally agreed therapeutic guidelines. The Tardieu Scale is not routinely used because it can be time-consuming and requires multiple notes. This study used the modified Ashworth scale and goniometry instead; however, the component of the range of motion that was affected or benefited after BoNT-A treatment was unknown. Thus, the effects observed may not have been specific to BoNT-A treatment. This may explain the absence of correlation between pain improvement and goniometry improvement. A number of potential confounders should be acknowledged. The complex patients included in the study had a number of conditions that could potentially influence response to therapy (i.e., depression, anxiety, cognitive disorders, type of brain injury), and a high proportion of the participants were also using nondrug therapies to support their rehabilitation. It should be stressed that patients with chronic post-stroke spasticity who received physical therapy were already receiving this treatment before BoNT-A injection and were functionally stable, suggesting that the observed pain reduction was associated with BoNT-A treatment. The use of antispasmodic treatments was permissible, and these may also affect response [40]. The potential for these interventions to influence response to aboBoNT-A treatment cannot be excluded, but given that any improvements were being observed on average 20 months post-stroke, it is possible that any benefits associated with these supportive therapies had been achieved prior to initiation of aboBoNT-A. The time that had elapsed since the stroke also reduces the likelihood that the benefits observed could be explained by spontaneous recovery.

Additionally, because patients are often aphasic following a stroke, a description of pain characteristics and intensity would have been compromised; almost one-fifth of the patients in the present study had significantly or completely impaired communication, and in such cases, pain evaluation was based on involuntary signs, such as grimacing and withdrawal, or caregiver reporting. The high rate of cognitive dysfunction may also have affected the ability of individuals to discuss their goals and describe their pain level; however, this was not considered to be severe enough to impact the findings of the study.

Despite the limitations associated with the observational nature of the study, by describing the muscles injected, the doses used, and the benefits achieved with BoNT-A in this chronic population, the BCause Study provides valuable insights in an area in which randomized controlled trials are still to be conducted.

## 4. Conclusions

Overall, these data suggest that aboBoNT-A may be a useful treatment for patients with chronic post-stroke spasticity who set shoulder pain as a therapeutic goal, and that further investigation in prospective, randomized trials is warranted. Treatment with aboBoNT-A injections in this population may provide clinical benefit to patients by reducing pain and improving ROM and improving the quality of patients’ and caregivers’ lives through increased independence of patients. Despite the prolonged duration between the onset of stroke/spasticity and the initiation of treatment in this study, repeated aboBoNT-A injections resulted in improved effectiveness with a durable response. These findings are particularly meaningful for patients with shoulder pain who have limited arm function, as well as their caregivers who may experience distress if activities that they assist with cause or worsen pain.

## 5. Materials and Methods

### 5.1. Study Design

The BCause Study was an observational, national, multicenter, noninterventional, postmarketing, prospective study (NCT02390206) conducted at 11 centers in Brazil between June 2015 and August 2017. Full details of the study design have been published previously [25].

Briefly, patients with upper limb spasticity, with or without lower limb involvement, received BoNT-A injections as part of routine treatment; thus, the investigators were free to choose the targeted muscles, BoNT-A preparation, injected doses, injection interval, number of injection points, and volume/dosage per point, in accordance with the local summary of product characteristics and locally agreed therapeutic guidelines.

Patients attended two visits in cycle 1 at baseline (visit 1) and months 3–6 (visit 2), and one visit in cycle 2 at months 6–12 (visit 3). All visits included clinical examination (ROM, MAS score, pain score (VNS), Barthel Index, QoL (EQ-5D-5L)) [33,41,42,43,44] and setting/review of treatment goals (GAS) [45,46].

The patients included in this post hoc analysis received aboBoNT-A only.

### 5.2. Participants

Full details of the inclusion and exclusion criteria for the BCause Study have been published previously [25]. Adult patients (18–80 years) with a last documented stroke (hemorrhagic or ischemic) at least 1 year prior to study entry were eligible for the study. All patients had documented upper limb spasticity, with or without lower limb spasticity. Patients who had received prior injections of BoNT-A to treat spasticity symptoms were excluded, as were those who had undergone previous surgical procedures for spasticity treatment, had received previous phenol injections, or had been indicated to receive phenol during the study. Patients with contraindications to any BoNT-A preparations were excluded.

Patients with upper limb spasticity following a stroke occurring at least 1 year prior to study entry, who had received at least one injection of BoNT-A and had one post-treatment assessment of their primary goal, were assessed in the BCause Study.

This post hoc analysis included aboBoNT-A-naive patients treated with aboBoNT-A who set improvement in shoulder pain as a primary or secondary treatment goal at cycle 1 and/or 2, and patients who received aboBoNT-A injections to the shoulder muscles in the same cycle. Patients with a primary or secondary GAS goal of pain not related to shoulder pain were excluded from this post hoc analysis.

Treatment in cycle 1 was administered at visit 1. Patients received aboBoNT-A injections to one or more of the following muscles: deltoideus, pectoralis major, subclavius, subscapularis, teres major, latissimus dorsi, biceps brachii, triceps brachii, and trapezius.

At the end of cycle 1 (visit 2), goal attainment was evaluated, goals were reset, and the second cycle of aboBoNT-A injections was administered. At the end of cycle 2 (visit 3), goal attainment was evaluated. Details on AboBoNT-A injections were not collected at visit 3.

### 5.3. Endpoints

The proportion of the responders (achievement or overachievement of the set pain goal (GAS of 0, +1, or +2) at the end of the considered injection cycle) at visits 2 and 3, and change from baseline to visits 2 and 3 in total GAS T-score, pain intensity (pain score (VAS) at rest and at movement during daily activities), shoulder ROM (shoulder abduction angle), shoulder MAS scores, QoL (EQ VAS) score, and the pain/discomfort domain of EQ-5D-5L were assessed.

The degree of independence reported by the patients was also assessed (Barthel Index score from baseline and change by visit 3).

The time interval between the first and second treatment was reported.

The physical and psychological burden of the disease was assessed using EQ-5D-5L for patients’ QoL, and a five-point Likert scale was used for the patients’ and caregivers’ overall satisfaction with treatment effectiveness.

### 5.4. Statistical Analysis

Descriptive statistics (frequency count and percentages of categorical variables, number of observations, mean, SD, median, minimum, and maximum) are presented for all assessments based on available data. The first and third quartiles, and 95% CI of the mean (or of the proportion) are presented as appropriate. Missing data were not imputed.

Correlations between change from baseline in pain score at movement and change from baseline in shoulder MAS score, pain score, ROM, and EQ VAS score were calculated at visits 2 and 3 using the Spearman correlation coefficient.

## Figures and Tables

**Table 1:** Baseline demographics and treatment information.

	Patients (N = 49)	
Female, n (%)	25 (51.0)	
Age, mean (SD) years	60.3 (9.1)	
Handedness, n (%)	Left 5 (10.2)	Right 44 (89.8)
Time since last CVA, median (range) months	20.6 (12–240)	
Time since onset of CVA, median (range) months	21.2 (12–240)	
Time since onset of spasticity, median (range) months (n)	16.1 (0–193) (47)]	
Time between first CVA and onset of spasticity, median (range) months (n)	4.0 (0–237) (42)]	
Upper limbs affected, n (%)	49 (100.0)	
Laterality, n (%)	Left 30 (61.2)	Right 18 (36.7)
	Bilateral 1 (2.0)	
Post-stroke complications, n (%) Cardiovascular disease Hypertension Hypercholesterolemia Pressure ulcer Urinary tract infection Bronchopulmonary infections Sleep disorder Loss of vision Depression/anxiety Dizziness Aphasia Fractures Falls	35 (71.4) 5 (14.3) 26 (74.3) 13 (37.1) 1 (2.9) 2 (5.7) 3 (8.6) 6 (17.1) 5 (14.3) 14 (40.0) 3 (8.6) 10 (28.6) 2 (5.7) 7 (20.0)	
Impairment of communication, n (%) None Mild Significant Total Not done	24 (49.0) 8 (16.3) 7 (14.3) 2 (4.1) 8 (16.3)	
Patients who had other conditions that could affect functional outcome, n (%) ^1^ Mood/emotional function Behavioral problems Fatigue Orientation Memory Attention	30 (61.2) 24 (49.0) 4 (13.3) 9 (30.0) 8 (26.7) 16 (53.3) 13 (43.3)	
Patients who underwent nondrug therapies, n (%) ^1^ Splinting Orthotics Physical therapies Home exercises Electrical stimulation	43 (87.8) 16 (33.3) 19 (39.6) 34 (70.8) 29 (60.4) 6 (12.5)	

^1^ More than one response was permissible; therefore, the sum of the percentages may exceed 100%. CVA, cerebrovascular accident; N, total number of patients; n, number of patients; SD, standard deviation.

**Table 2:** AbobotulinumtoxinA injections to shoulder muscles during two cycles of treatment.

	Patients (N = 49)
Total dose administered in upper limb median (range) units (n) Visit 1 (start of cycle 1) Visit 2 (start of cycle 2)	600.0 (100.0–1200.0) (49) 600.0 (100.0–1500.0) (46) ^1^
Number of muscles injected in upper limb mean (SD) (n) Visit 1 (start of cycle 1) Visit 2 (start of cycle 2)	5.8 (2.6) (49) 5.7 (2.5) (46) ^1^
Muscles injected, n (%) Visit 1 (start of cycle 1) Pectoralis major Latissimus dorsi Subscapularis Teres major Visit 2 (start of cycle 2) Pectoralis major Latissimus dorsi Subscapularis Teres major	*n* = 49 44 (89.8) 27 (55.1) 13 (26.5) 1 (2.0) N = 46 ^1^ 35 (76.1) 26 (56.5) 12 (26.1) 1 (2.2)
Injection guidance technique used, n (%) ^2^ Visit 1 (start of cycle 1) Palpation/anatomic landmarks Electrical stimulation Visit 2 (start of cycle 2) Palpation/anatomic landmarks Electrical stimulation	*n* = 49 41 (83.7) 9 (18.4) *n* = 46 ^1^ 39 (84.8) 9 (19.6)
Time in months between first and second treatment mean (SD)	*n* = 46 4.63 (0.74)

^1^ One patient received abobotulinumtoxinA injections to lower limbs only at visit 2. ^2^ Both techniques may have been used; therefore, the sum of the percentages may exceed 100%. N, total number of patients; n, number of patients; SD, standard deviation.

**Table 3:** Quality-of-life assessments at baseline, end of cycle 1 (visit 2), and end of cycle 2 (visit 3).

	Baseline	Visit 2	Visit 3	Change from Baseline to Visit 3
Barthel Index score, mean (SD) (n)	56.5 (28.1) (49)	NA	65.8 (27.8) (42)	4.3 (10.5) (42)
EQ VAS score, mean (SD) (n)	51.0 (26.4) (31)	59.6 (26.7) (25)	64.7 (24.1) (23)	16.7 (28.7) (23)
Pain/discomfort, n (%) ^1^ No pain Slight Moderate Severe Extreme Missing	3 (9.7) 7 (22.6) 12 (38.7) 5 (16.1) 4 (12.9) 18	6 (24.0) 10 (40.0) 5 (20.0) 3 (12.0) 1 (4.0) 24	9 (39.1) 9 (39.1) 3 (13.0) 2 (8.7) 0 26	NA

^1^ Percentages are based on the number of nonmissing observations. EQ VAS, EuroQol visual analogue scale; NA, not applicable; SD, standard deviation.

## Data Availability

Qualified researchers may request access to patient-level study data that underlie the results reported in this publication. Additional relevant study documents, including the clinical study report, study protocol with any amendments, annotated case report form, statistical analysis plan, and dataset specifications may also be made available. Patient-level data will be anonymized, and study documents will be redacted to protect the privacy of the study participants. Where applicable, data from eligible studies are available 6 months after the studied medicine and indication have been approved in the US and EU or after the primary manuscript describing the results has been accepted for publication, whichever is later. Further details on Ipsen’s sharing criteria, eligible studies, and process for sharing are available here (https://vivli.org/members/ourmembers/ accessed 11 August 2022). Any requests should be submitted to www.vivli.org for assessment by an independent scientific review board.

## Abbreviations

aboBoNT-A: abobotulinumtoxinA; BCause: botulinum toxin in the treatment of chronic post-stroke spastic patients; BoNT-A: botulinum toxin A; CVA: cerebrovascular accident; EQ-5D-5L: five-dimension five-level EuroQol questionnaire; GAS: goal attainment scaling; GAS T: total goal attainment scaling; MAS: modified Ashworth scale; ROM: range of motion; ULIS: Upper Limb International Study; VNS: visual numeric scale.

## References

[1] Viana R., Pereira S., Mehta S., Miller T., Teasell R. (2012). Evidence for therapeutic interventions for hemiplegic shoulder pain during the chronic stage of stroke: A review. Top. Stroke Rehabil..

[2] Walsh K. (2001). Management of shoulder pain in patients with stroke. Postgrad. Med. J..

[3] Gamble G.E., Barberan E., Bowsher D., Tyrrell P.J., Jones A.K. (2000). Post stroke shoulder pain: More common than previously realized. Eur. J. Pain.

[4] Gamble G.E., Barberan E., Laasch H.U., Bowsher D., Tyrrell P.J., Jones A.K. (2002). Poststroke shoulder pain: A prospective study of the association and risk factors in 152 patients from a consecutive cohort of 205 patients presenting with stroke. Eur. J. Pain.

[5] Lindgren I., Jönsson A.C., Norrving B., Lindgren A. (2007). Shoulder pain after stroke: A prospective population-based study. Stroke.

[6] Turner-Stokes L., Jackson D. (2002). Shoulder pain after stroke: A review of the evidence base to inform the development of an integrated care pathway. Clin. Rehabil..

[7] Nickel R., Lange M., Stoffel D.P., Navarro E.J., Zetola V.F. (2017). Upper limb function and functional independence in patients with shoulder pain after stroke. Arq. Neuro-Psiquiatr..

[8] Yang S., Chang M.C. (2021). Poststroke Pain. Semin. Neurol..

[9] Dysport Prescribing Information. https://www.accessdata.fda.gov/drugsatfda_docs/label/2019/125274s115lbl.pdf.

[10] Dysport Summary of Product Characteristics. https://www.medicines.org.uk/emc/product/7261/smpc.

[11] BOTOX Prescribing Information. https://www.rxabbvie.com/pdf/botox_pi.pdf.

[12] BOTOX Summary of Product Characteristics. https://www.medicines.org.uk/emc/product/859/smpc#gref.

[13] Chang K.V., Chiu Y.H., Wu W.T., Hsu P.C., Özçakar L. (2020). Botulinum toxin injections for shoulder and upper limb pain: A narrative review. Pain Manag..

[14] Wissel J., Müller J., Dressnandt J., Heinen F., Naumann M., Topka H., Poewe W. (2000). Management of spasticity associated pain with botulinum toxin A. J. Pain Symptom Manag..

[15] Jabbari B. (2008). Botulinum neurotoxins in the treatment of refractory pain. Nat. Clin. Pract. Neurol..

[16] Oh H.M., Chung M.E. (2015). Botulinum Toxin for Neuropathic Pain: A Review of the Literature. Toxins.

[17] Winner B.M., Bodt S.M.L., McNutt P.M. (2020). Special Delivery: Potential Mechanisms of Botulinum Neurotoxin Uptake and Trafficking within Motor Nerve Terminals. Int. J. Mol. Sci..

[18] Alvisi E., Serrao M., Conte C., Alfonsi E., Tassorelli C., Prunetti P., Cristina S., Perrotta A., Pierelli F., Sandrini G. (2018). Botulinum toxin A modifies nociceptive withdrawal reflex in subacute stroke patients. Brain Behav..

[19] Luvisetto S. (2020). Botulinum Toxin and Neuronal Regeneration after Traumatic Injury of Central and Peripheral Nervous System. Toxins.

[20] Yelnik A.P., Colle F.M., Bonan I.V., Vicaut E. (2007). Treatment of shoulder pain in spastic hemiplegia by reducing spasticity of the subscapular muscle: A randomised, double blind, placebo controlled study of botulinum toxin A. J. Neurol. Neurosurg. Psychiatry.

[21] Wu T., Fu Y., Song H.X., Ye Y., Dong Y., Li J.H. (2015). Effectiveness of Botulinum Toxin for Shoulder Pain Treatment: A Systematic Review and Meta-Analysis. Arch. Phys. Med. Rehabil..

[22] Pedreira G., Cardoso E., Melo A. (2008). Botulinum toxin type A for refractory post-stroke shoulder pain. Arq. Neuro-Psiquiatr..

[23] Lejeune T., Khatkova S., Turner-Stokes L., Picaut P., Maisonobe P., Balcaitiene J., Boyer F.C. (2020). Abobotulinumtoxina injections in shoulder muscles to improve adult upper limb spasticity: Results from a phase 4 real-world study and a phase 3 open-label trial. J. Rehabil. Med..

[24] Yan L.L., Li C., Chen J., Miranda J.J., Luo R., Bettger J., Zhu Y., Feigin V., O’Donnell M., Zhao D. (2016). Prevention, management, and rehabilitation of stroke in low- and middle-income countries. eNeurologicalSci.

[25] Khan P., Riberto M., Frances J.A., Chueire R., Amorim A., Xerez D., Chung T.M., Mercuri L.H.C., Longo A.L., Lianza S. (2020). The Effectiveness of Botulinum Toxin Type A (BoNT-A) Treatment in Brazilian Patients with Chronic Post-Stroke Spasticity: Results from the Observational, Multicenter, Prospective BCause Study. Toxins.

[26] Crema C.M.T., Santos A.P.B.C., Magário L.P.T., Caldas C.A.C.T., Riberto M. (2016). Neuromuscular block practice in the treatment of spasticity in Brazil. Acta Fisiátrica.

[27] Fitterer J.W., Picelli A., Winston P. (2021). A Novel Approach to New-Onset Hemiplegic Shoulder Pain with Decreased Range of Motion Using Targeted Diagnostic Nerve Blocks: The ViVe Algorithm. Front. Neurol..

[28] Liporaci F.M., Mourani M.M., Riberto M. (2019). The myofascial component of the pain in the painful shoulder of the hemiplegic patient. Clinics.

[29] Kalichman L., Ratmansky M. (2011). Underlying pathology and associated factors of hemiplegic shoulder pain. Am. J. Phys. Med. Rehabil..

[30] Gomes A.L.S., Mello F.F., Cocicov Neto J., Benedeti M.C., Modolo L.F.M., Riberto M. (2019). Can the positions of the spastic upper limb in stroke survivors help muscle choice for botulinum toxin injections?. Arq. Neuro-Psiquiatr..

[31] Alter K.E., Karp B.I. (2017). Ultrasound Guidance for Botulinum Neurotoxin Chemodenervation Procedures. Toxins.

[32] Elovic E.P., Esquenazi A., Alter K.E., Lin J.L., Alfaro A., Kaelin D.L. (2009). Chemodenervation and nerve blocks in the diagnosis and management of spasticity and muscle overactivity. PMR.

[33] Turner-Stokes L., Fheodoroff K., Jacinto J., Maisonobe P. (2013). Results from the Upper Limb International Spasticity Study-II (ULIS-II): A large, international, prospective cohort study investigating practice and goal attainment following treatment with botulinum toxin A in real-life clinical management. BMJ Open.

[34] Turner-Stokes L., Jacinto J., Fheodoroff K., Brashear A., Maisonobe P., Lysandropoulos A., Ashford S. (2021). Assessing the effectiveness of upper-limb spasticity management using a structured approach to goal-setting and outcome measurement: First cycle results from the ULIS-III Study. J. Rehabil. Med..

[35] Wissel J., Fheodoroff K., Hoonhorst M., Müngersdorf M., Gallien P., Meier N., Hamacher J., Hefter H., Maisonobe P., Koch M. (2020). Effectiveness of AbobotulinumtoxinA in Post-stroke Upper Limb Spasticity in Relation to Timing of Treatment. Front. Neurol..

[36] Gracies J.M., O’Dell M., Vecchio M., Hedera P., Kocer S., Rudzinska-Bar M., Rubin B., Timerbaeva S.L., Lusakowska A., Boyer F.C. (2018). Effects of repeated abobotulinumtoxinA injections in upper limb spasticity. Muscle Nerve.

[37] Marco E., Duarte E., Vila J., Tejero M., Guillen A., Boza R., Escalada F., Espadaler J.M. (2007). Is botulinum toxin type A effective in the treatment of spastic shoulder pain in patients after stroke? A double-blind randomized clinical trial. J. Rehabil. Med..

[38] Ghroubi S., Alila S., Elleuch W., Ayed H.B., Mhiri C., Elleuch M.H. (2020). Efficacy of botulinum toxin A for the treatment of hemiparesis in adults with chronic upper limb spasticity. Pan Afr. Med. J..

[39] Carod-Artal F.J., Medeiros M.S., Horan T.A., Braga L.W. (2005). Predictive factors of functional gain in long-term stroke survivors admitted to a rehabilitation programme. Brain Inj..

[40] Creamer M., Cloud G., Kossmehl P., Yochelson M., Francisco G.E., Ward A.B., Wissel J., Zampolini M., Abouihia A., Calabrese A. (2018). Effect of Intrathecal Baclofen on Pain and Quality of Life in Poststroke Spasticity. Stroke.

[41] Golicki D., Niewada M., Buczek J., Karlińska A., Kobayashi A., Janssen M.F., Pickard A.S. (2015). Validity of EQ-5D-5L in stroke. Qual. Life Res..

[42] Gracies J.M., Brashear A., Jech R., McAllister P., Banach M., Valkovic P., Walker H., Marciniak C., Deltombe T., Skoromets A. (2015). Safety and efficacy of abobotulinumtoxinA for hemiparesis in adults with upper limb spasticity after stroke or traumatic brain injury: A double-blind randomised controlled trial. Lancet Neurol..

[43] Gracies J.M., Esquenazi A., Brashear A., Banach M., Kocer S., Jech R., Khatkova S., Benetin J., Vecchio M., McAllister P. (2017). Efficacy and safety of abobotulinumtoxinA in spastic lower limb: Randomized trial and extension. Neurology.

[44] Quinn T.J., Langhorne P., Stott D.J. (2011). Barthel index for stroke trials: Development, properties, and application. Stroke.

[45] Turner-Stokes L. (2009). Goal attainment scaling (GAS) in rehabilitation: A practical guide. Clin. Rehabil..

[46] Turner-Stokes L. (2013). Upper limb international spasticity study: Rationale and protocol for a large, international, multicentre prospective cohort study investigating management and goal attainment following treatment with botulinum toxin A in real-life clinical practice. BMJ Open.

